# Large café-au-lait spots on a 5-year-old boy

**DOI:** 10.1016/j.jdcr.2022.08.025

**Published:** 2022-08-27

**Authors:** Brett D. McLarney, Jennifer J. Parker, Sylvia Hsu

**Affiliations:** Department of Dermatology, Temple University Lewis Katz School of Medicine, Philadelphia, Pennsylvania

**Keywords:** café-au-lait, endocrinopathy, fibrous dysplasia, MAS, McCune-Albright syndrome, NF1, neurofibromatosis 1

## Case

**Fig 1:**
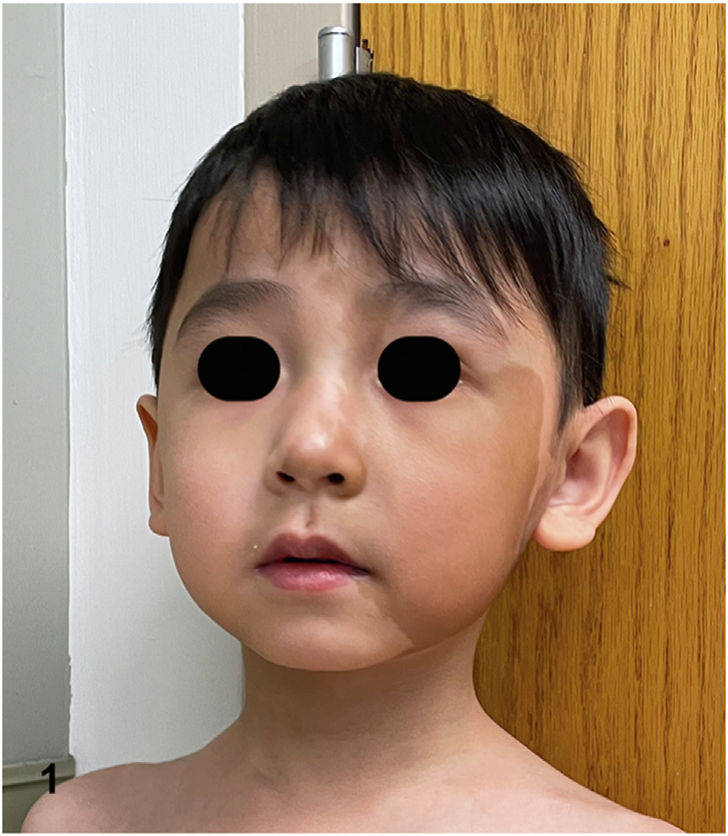

**Fig 2:**
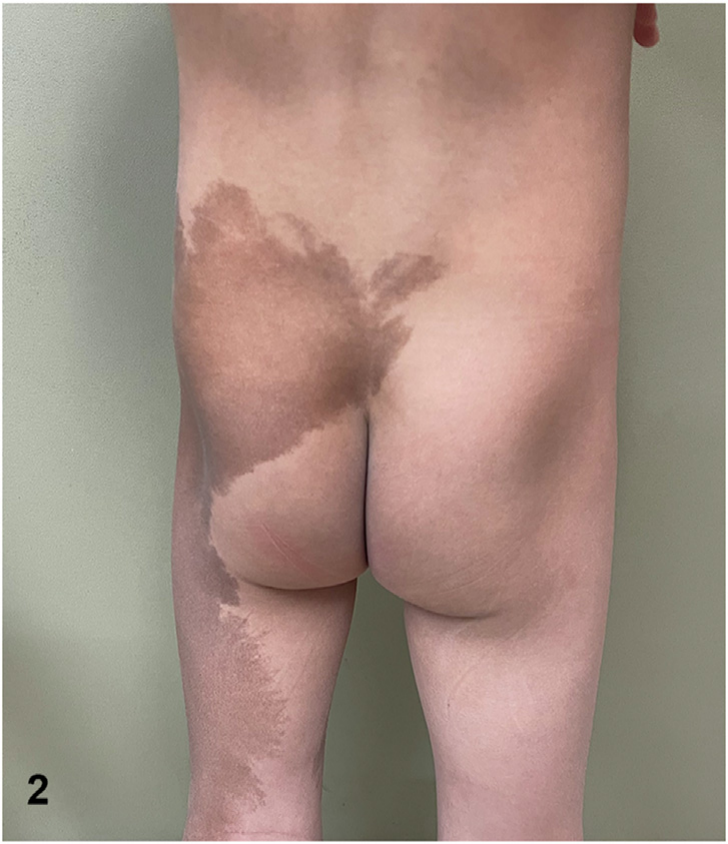

A 5-year-old boy presented for cosmetic concerns regarding an irregularly shaped, café-au-lait patch on his left cheek (Fig 1). Further visual inspection revealed an additional, large café-au-lait spot with a jagged border extending from the patient’s left buttock to distal leg (Fig 2). Both patches were present since birth. Plain radiographs of the patient's long bones demonstrated fibrous dysplasia of the proximal femurs bilaterally with lesions measuring up to 3.1 cm × 1.5 cm on the right and 6.4 cm × 3.4 cm on the left. Basic laboratory workup including complete blood count with differential test, comprehensive metabolic panel, and C-reactive protein revealed no abnormalities.

**Question 1: What is the most likely diagnosis?**

A. Tuberous sclerosis
B. Neurofibromatosis 1
C. Neurocutaneous melanosis
D. McCune-Albright syndrome
E. Congenital dermal melanocytosis

**Answer:**

A. Tuberous sclerosis – Incorrect. Neither café-au-lait spots nor fibrous dysplasia is characteristic of tuberous sclerosis.
B. Neurofibromatosis 1 – Incorrect. While patients with neurofibromatosis 1 (NF1) can experience café-au-lait spots and long bone dysplasia, the café-au-lait spots of NF1 have a smooth “coast of California” border. This is to be contrasted with the jagged “coast of Maine” border characteristic of the café-au-lait spots observed in McCune-Albright syndrome (MAS). Respect for the midline is another distinctive feature of this patient's café-au-lait spots that favors association with MAS over NF1 and other conditions manifesting with café-au-lait spots.
C. Neurocutaneous melanosis – Incorrect. Neurocutaneous melanosis is characterized by a congenital melanocytic nevus with associated neuromelanosis. While some melanocytic nevi can visually resemble the café-au-lait spots of MAS, neuromelanosis is associated with a melanocytic proliferation within the leptomeninges and brain parenchyma and not associated with polyostotic fibrous dysplasia.
D. McCune-Albright syndrome – Correct. MAS is a rare disorder characterized by a triad of café-au-lait spots with jagged “coast of Maine” borders, polyostotic fibrous dysplasia, and endocrine dysfunction. The diagnosis can be made clinically as long as 2 features of this triad are observed. Endocrine abnormalities most often take the form of precocious puberty, thyroid dysfunction, or growth hormone excess. For more information regarding laboratory testing and imaging to be performed in suspected and newly diagnosed MAS patients, see the referenced consensus guidelines.^1^
E. Congenital dermal melanocytosis – Incorrect. Congenital dermal melanocytosis is characterized by a patch, that is more blue or gray in color than this patient’s café-au-lait spots.

**Question 2: In addition to skeletal problems, this patient is at highest risk for which of the following?**

A. Precocious puberty
B. Seizures
C. Optic glioma
D. Gastrointestinal neoplasia
E. Infertility

**Answer:**

A. Precocious puberty – Correct. Precocious puberty itself was formerly a component of the MAS triad, before thyroid dysfunction and growth hormone excess, among other endocrinopathies, were recognized as typical manifestations of the disease. Precocious puberty in males with MAS most often manifests as testicular enlargement, appearance of axillary and pubic hair, and increased body odor. Additionally, renal involvement as manifested by phosphate wasting is another common feature of patients with MAS.^1^
B. Seizures – Incorrect. Port-wine stains involving the forehead can be associated with Sturge-Weber syndrome in which patients are at risk for seizures. Although the café-au-lait spot of this patient involves the forehead, it is not a port-wine stain and he is not at increased risk for seizures.
C. Optic glioma – Incorrect. The incidence of optic gliomas among patients with NF1 is 15% to 20%.^2^ On the other hand, patients with MAS are not at increased risk.
D. Gastrointestinal neoplasia – Incorrect. Patients with certain conditions such as Peutz-Jehgers syndrome and Cowden disease are at increased risk for gastrointestinal polyps and gastrointestinal adenocarcinoma, but this is not the case for patients with MAS.
E. Infertility – Incorrect. Female patients with MAS are at increased risk for menstrual disturbance and infertility. However, infertility occurs secondarily to precocious puberty and menstrual irregularity; furthermore, this patient is a male.^3^

**Question 3: Which term best describes the GNAS gene mutation of McCune-Albright syndrome?**

A. Autosomal dominant
B. X-linked recessive
C. Anticipation
D. Autosomal recessive
E. Somatic mutation

**Answer:**

A. Autosomal dominant – Incorrect. The mutation in *GNAS* that is associated with MAS is a somatic mutation that occurs after fertilization. MAS is commonly confused with NF1, which is inherited in an autosomal dominant pattern.
B. X-linked recessive – Incorrect. X-linked ichthyosis, X-linked reticulate pigmentary disorder, and hypohidrotic ectodermal dysplasia are examples of X-linked dermatologic diseases. *GNAS* is located on chromosome 20, not the X-chromosome.^4^
C. Anticipation – Incorrect. Anticipation is a feature of many trinucleotide repeat disorders. MAS is not a trinucleotide repeat disorder.
D. Autosomal recessive – Incorrect. The *GNAS* mutations associated with MAS are somatic and not germline; therefore, these mutations are not inherited.
E. Somatic mutation – Correct. In MAS, a mutation to the *GNAS* gene occurs that is a somatic missense gain-of-function mutation at Arg201 or Gln227. It occurs after fertilization, resulting in genetic mosaicism; germline involvement of such gain-of-function mutations is presumed to be incompatible with life as MAS has not been observed to be inherited. The presentation and natural course of MAS are variable and dependent on which cells incurred that *GNAS* mutation. *GNAS* encodes the cyclic adenosine monophosphate-regulating protein subunit, Gsα, and its mutation in MAS causes a constitutively active cyclic adenosine monophosphate pathway in the affected cells. In the skin, Gsα mediates the action of alpha-melanocyte-stimulating hormone to stimulate melanin production, resulting in the characteristic café-au-lait spots of MAS.^5^

## Conflicts of interest

None disclosed.
